# White Blood Cell Count in Women: Relation to Inflammatory Biomarkers, Haematological Profiles, Visceral Adiposity, and Other Cardiovascular Risk Factors

**DOI:** 10.3329/jhpn.v31i1.14749

**Published:** 2013-03

**Authors:** Mahdieh Abbasalizad Farhangi, Seyyed-Ali Keshavarz, Mohammadreza Eshraghian, Alireza Ostadrahimi, Ali-Akbar Saboor-Yaraghi

**Affiliations:** ^1^Department of Community Nutrition, School of Health and Nutrition, Tabriz University of Medical Sciences, Tabriz, Iran;; ^2^Department of Cellular and Molecular Nutrition, School of Nutritional Sciences and Dietetics, Tehran University of Medical Sciences, Tehran, Iran; ^3^Department of Nutrition and Biochemistry, School of Public Health, Tehran University of Medical Sciences, Tehran, Iran; ^4^Department of Biostatistics and Epidemiology, School of Public Health, Tehran University of Medical Sciences, Tehran, Iran;; ^5^Nutrition Research Center, School of Nutrition, Tabriz University of Medical Sciences, Tabriz, Iran

**Keywords:** Angiotensin Π, C-reactive protein, Interleukin 6, Obesity, White blood cell count

## Abstract

The role of white blood cell (WBC) count in pathogenesis of diabetes, cardiovascular disease, and obesity-related disorders has been reported earlier. Recent studies revealed that higher WBC contributes to atherosclerotic progression and impaired fasting glucose. However, it is unknown whether variations in WBC and haematologic profiles can occur in healthy obese individuals. The aim of this study is to further evaluate the influence of obesity on WBC count, inflammatory biomarkers, and metabolic risk factors in healthy women to establish a relationship among variables analyzed. The sample of the present study consisted of 84 healthy women with mean age of 35.56±6.83 years. They were categorized into two groups based on their body mass index (BMI): obese group with BMI >30 kg/m^2^ and non-obese group with BMI <30 kg/m^2^. We evaluated the relationship between WBC and platelet count (PLT) with serum interleukin 6 (IL-6), C-reactive protein (CRP), angiotensin Π (Ang Π), body fat percentage (BF %), waist-circumference (WC), and lipid profile. WBC, PLT, CRP, and IL-6 in obese subjects were significantly higher than in non-obese subjects (p< 0.05). The mean WBC count in obese subjects was 6.4±0.3 (*×*10^9^/L) compared to 4.4±0.3 (*×*10^9^/L) in non-obese subjects (p=0.035). WBC correlated with BF% (r=0.31, p=0.004), CRP (r=0.25, P=0.03), WC (r=0.22, p=0.04), angiotensin Π (r=0.24, p=0.03), triglyceride (r=0.24, p=0.03), and atherogenic index of plasma (AIP) levels (r=0.3, p=0.028) but not with IL-6. Platelet count was also associated with WC and waist-to-hip ratio (p<0.05). Haemoglobin and haematocrit were in consistent relationship with LDL-cholesterol (p<0.05). In conclusion, obesity was associated with higher WBC count and inflammatory parameters. There was also a positive relationship between WBC count and several inflammatory and metabolic risk factors in healthy women.

## INTRODUCTION

Recent epidemiological studies revealed a positive relationship between cardiovascular disease and inflammatory markers (1). White blood cell (WBC) count, as one of the major components of inflammatory process, plays an important role in pathogenesis of insulin resistance and cardiovascular disease (2). The positive relationship between WBC count, insulin resistance, hypertension, and cardiovascular disease has been observed in several studies (1-3) whereas it is unknown whether these variations can occur in healthy obese individuals. Pro-inflammatory cytokines, such as interleukin 6 (IL-6) and interleukin 8 (IL-8), are important inducers of WBC production (4). It can be speculated that the elevated amount of these cytokines is responsible for higher WBC count in diabetes or cardiovascular disease. Adipose tissue is a great source of inflammatory factors, such as IL-6 and C-reactive protein (CRP), which also are well-established markers of systemic inflammation (5). Certainly, there has been an enormous interest in the identification of the relationship between WBC count and these inflammatory factors in obese persons with higher fat storages, who are at greater risk of hypertension or complicating cardiovascular disorders (3). Angiotensin Π has recently been proposed as a novel pro-inflammatory mediator released by adipose tissue and plays an important role in vasoconstriction and production of inflammatory factors, such as IL-6, IL-8, and vascular cell adhesion molecule (6,7). To our knowledge, no studies have yet evaluated the relationship of cytokines, such as IL-6, CRP, and angiotensin Π, with WBC and haematological parameters in obesity. Therefore, in this case-control study, we investigated the relationship between WBC count, haematological profile, and inflammatory factors, including CRP, IL-6, and angiotensin Π with fat mass, waist-circumference, and metabolic risk factors of cardiovascular disease in women.

## MATERIALS AND METHODS

### Study subjects

We examined 84 healthy volunteer women aged 35.56±6.83 years. The subjects were recruited by invitation letters distributed in different parts of Tabriz city. All subjects provided written informed consent prior to participation in the study. Demographic and medical characteristics of subjects were obtained through appropriate questionnaire. The questionnaire included information about age, marital status, smoking habits, medication, and disease status. Information on physical activity was obtained through International Physical Activity Questionnaire (IPAQ) (8). Exclusion criteria of the study subjects were as follows: taking any hormone-replacement therapy, history of hypertension, thyroid dysfunction, hepatic or renal disease, any disease known to influence immune system, and being pregnant or lactating. The research protocol was approved by the Ethics Committee of Human Experimentation of the Tehran University of Medical Sciences (TUMS-Project number 89-04-27-11869).

### Anthropometric parameters

Weight was measured by calibrated Seca scale (Itin Scale Co., Inc., Germany) with the precision of 0.1 g and height by a cotton ruler. BMI was calculated as weight (kg)/(height in m)^2^. The waist-circumference was measured above the iliac crest at the natural waistline, and the hip measurement was taken at the largest area of the natural hipline. Waist-to-hip ratio (WHR) was also calculated based on waist and hip measurements. Body fat was measured by bioelectrical impedance analysis (Human-IM Plus; DS Dietosystem, Milan, Italy).

### Biochemical parameters

After an overnight fasting (10-12 hours), venous blood samples (10.0 mL) were collected at 8:30-9:30 am in the morning. Three mL of blood samples was immediately transferred to ethylendiaminetetraacetate (EDTA) specimen bottles for measurement of haematological parameters. The remaining blood was put into a dry tube for serum extraction and analysis of biochemical profile. Total and differential WBC and haematologic profiles were determined using an automatic cell counter (Drew Scientific, Excell-18; Minnesota, USA). Blood sugar was determined by glucose oxidase-peroxidase (GOD-POD) method, using a kit (Pars-Azmoon; Tehran, Iran). Total cholesterol (TC), triglyceride (TG), and high-density lipoprotein cholesterol (HDL-C) were analyzed by enzymatic colorimetric method (Pars-Azmoon, Tehran, Iran). Serum low-density lipoprotein cholesterol (LDL-C) was determined by Friedwald formula: LDL-cholesterol=TC-(HDL+TG/5) (9). Atherogenic index of plasma (AIP) was defined as Log (Triglycerides/HDL-cholesterol) (10). High-sensitivity C-reactive protein (hs-CRP) was measured by ELISA method (DRG Instruments GmbH, Germany). The inter- and intra-assay coefficients of variation (CV) for hs-CRP were <4.1% and <7.5% respectively. IL-6 levels were assayed with a commercial enzyme-linked immunosorbent assay (Quantikine IL-6 Immunoassay; e-Bioscience, San Diego, CA, USA). Each assay was performed with recombinant IL-6 standards according to the manufacturer's protocol. This assay has a dynamic range of 2 to 200 pg/mL and a sensitivity of 4 pg/mL. Serum angiotensin Π was also measured by ELISA method (Enzo, Life Science Inc.). The inter- and intra-assay coefficients of variation (CV) for this assay were <7.5% and <7.3% respectively.

### Statistical analysis

The data were analyzed using standard statistical methods provided by SPSS software (version 17, SPSS Inc., Chicago, IL, USA). The Kolmogorov-Smirnov test was used for verifying the hypothesis of normal distribution; all data were normally distributed, except in the case of IL-6. Independent sample *t*-tests were used for comparing normally-distributed data and Mann-Whitney U-test for data not normally distributed. The relationship between variables was examined with Pearson's correlation test or Spearman's rank correlation test. Partial correlation analysis was performed to determine the predictor variables of WBC, independent of confounders. All descriptive data were expressed as mean±standard deviation (SD). A p value less than 0.05 was considered significant.

## RESULTS

**Figure 1.**
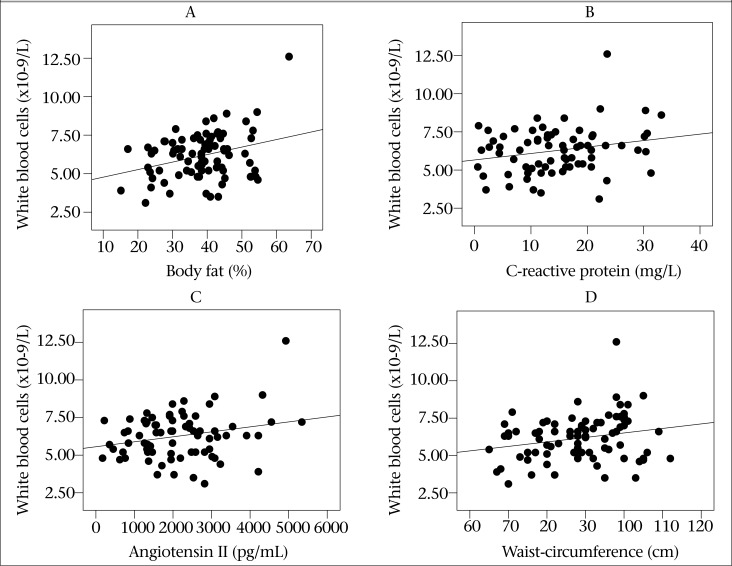
Relation of white blood cell count with selected anthropometric and inflammatory parameters (A: r=0.31, p=0.004; B: r=0.25, p=0.034; C: r=0.243, p=0.037; D: r=0.226, p=0.04)

**Figure 2.**
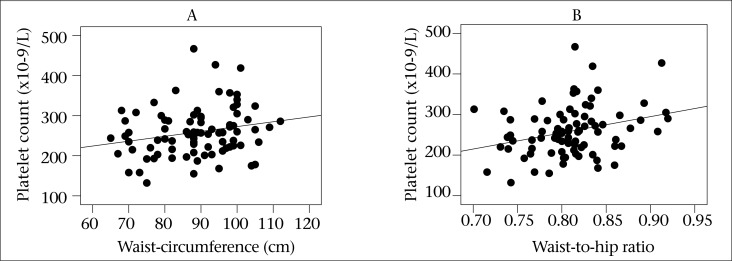
Relation between platelet count, WC, and WHR (A: r=0.22, p=0.04; B: r=0.29, r=0.008)

The main physical and biochemical features of the subjects are presented in Table 1. Serum FBS, TG, TC, and AIP in subjects with BMI >30 kg/m^2^ were significantly higher than those with BMI <30 kg/m^2^. In addition, obese subjects had significantly higher hs-CRP and IL-6 concentrations (p<0.05). Comparison of haematological parameters between groups is shown in Table 2. Significantly higher levels of WBC, lymphocytes, granulocytes, PLT and lower levels of haemoglobin (HGB) were observed in obese subjects compared to non-obese subjects. The WBC count showed a significant positive relationship with body fat (%), CRP, Ang Π, waist-circumference, and AIP (Figure 1). These relationships remained significant, even after adjusting for the confounding effect of physical activity (Table 3). Platelet count was also positively associated with WC and WHR (Figure 2). There was also a positive relationship between WBC, TG, and AIP and between LDL-C, HGB and haematocrit (HCT) (Table 4). These findings suggest that higher WBC and PLT count is related to higher risk of metabolic syndrome or atherogenic profile in obesity.

**T1able 1.** Physical and biochemical characteristics of study subjects (mean±SD)

Variable	BMI >30 (kg/m^2^)	BMI <30 (kg/m^2^)	p value
	(n=44)	(n=40)	
Age (years)	36.6±6.0	34.1±7.7	0.183
Weight (kg)	83.4±9.0	62.3±9.0	<0.001
Height (m)	1.6±0.1	1.6±0.0	NS
BMI (kg/m^2^)	33.7±3.4	24.5±3.5	<0.001
WC (cm)	96.3±6.7	79.5±8.4	<0.001
WHR	0.8±0.1	0.7±0.1	<0.001
Body fat (%)	44.2±7.4	31.9±7.1	<0.001
PA (Met-min/week)	1342.7±1504.4	1506.2±1122.6	NS
FBS (mmol/L)	4.5±0.9	4.2±0.1	0.03
TG (mmol/L)	1.6±0.6	1.3±0.5	<0.001
TC (mmol/L)	4.3±1.1	3.8±0.7	0.03
LDL-C (mmol/L)	2.1±0.9	1.2±0.6	NS
HDL-C (mmol/L)	1.4±0.2	1.3±0.1	NS
AIP	0.02±0.1	-0.04±0.1	<0.01
CRP (mg/L)	9.6±5.0	7.5±4.2	0.027
Ang Π (ng/mL)	2.0±1.0	2.2±1.2	NS
IL-6 (pg/mL)	30.1±20.0	28.9±27.6	0.047

AIP=Atherogenic index of plasma;

Ang Π=Angiotensin Π;

BMI=Body mass index;

CRP=C-reactive protein;

FBS=Fasting blood sugar;

HDL-C=High-density lipoprotein cholesterol;

IL-6=Interleukin 6;

LDL-C=Low-density lipoprotein cholesterol;

NS=Not significant;

PA=Physical activity;

TC=Total cholesterol;

TG=Triglyceride;

WC=Waist-circumference;

WHR=Waist-to-hip ratio

## DISCUSSION

This study demonstrates that total and differential white blood cell counts are higher in obese compared to non-obese women. Physical activity influences the circulating effect of inflammatory cytokines and WBC. Exercise in adults is associated with lower levels of C-reactive protein and WBC (11,12). In our study, WBC was positively associated with percentage of body fat, CRP, and angiotensin Π, independent of physical activity score. This suggested that correlation between WBC count and fat mass (13-16) could be mediated partially by inflammatory biomarkers. To our knowledge, it is the first report of WBC's relationship with inflammatory factors, especially angiotensin Π in obesity.

Adipose tissue functions as an endocrine organ with metabolic activities. Adipose tissue produces and releases a variety of inflammatory factors and adipokines as well as cytokines and chemokines, which have been implicated as active participants in the development of insulin resistance and cardiovascular disease (5). CRP, as an acute-phase protein which originates in the liver as well as adipose tissue, has many pathophysiologic roles in low-grade inflammation in obesity (17). WBC count increases in inflammation. Systemic low-grade inflammatory response in obesity is associated with higher WBC counts and two- to three-fold increase in the systemic concentrations of CRP and IL-6 (18). IL-6 by itself further increases the production of CRP in obesity (19,20). This elevated amount of CRP is taken up by WBC, promotes migration of WBC into arterial wall, and activates the complement system and foam cell production (21). An increased CRP level is in parallel of elevated WBC production, and this mechanism can partially explain the positive relationship between these two parameters.

**T1able 2.** Haematological parameters of study subjects

Variable	BMI >30 (kg/m^2^) (n=44)	BMI <30 (kg/m^2^) (n=40)	Normal range	p value
Total WBC (×10^9^/L)	6.4±0.3	4.4±0.3	4-11	0.035
Lymphocytes (×10^9^/L)	2.3±0.7	2.1±0.5	1-4	0.042
Granulocytes (×10^9^/L)	3.9±1.1	3.4±0.9	2-7.7	0.043
MID (×10^9^/L)	0.1±0.1	0.1±0.1	0.1-1.1	NS
RBC (×10^12^/L)	4.4±0.3	4.5±0.3	3.9–5.6	NS
HCT (Proportion of 1.0)	0.4±0.1	0.4±0.0	0.3-0.5	NS
HGB (g/L)	125.1±10.4	130.1±9.5	120-160	0.026
MCHC (g/L)	329.8±10.1	332.9±8.5	320-360	NS
MCV (fL)	86.2±8.7	87.9±5.7	82-98	NS
PLT(×10^9^/L)	271.4±61.4	244.6±59.3	140-400	0.047

HCT=Haematocrit;

HGB=Haemoglobin;

MCHC=Mean corpuscular haemoglobin concentration;

MCV=Mean corpuscular volume;

NS=Not significant;

PLT=Platelet count;

RBC=Red blood cell;

WBC=White blood cell;

MID=Minimum inhibitory dilution

We also found a positive relationship between WBC and angiotensin Π (p<0.05). This can also be explained by the pro-inflammatory role of this molecule; accumulating evidence suggests that angiotensin Π mediates its effects by increasing the pro-inflammatory cytokine production, such as IL-6 and IL-8 (6); these two cytokines are potent inducers of WBC production (4). It has been shown that inhibition of angiotensin Π production is associated with reduction in WBC count (22). More interestingly, the link between elevated WBC count and hypertension, which has been reported in previous studies (23,24), may be directly related to the inflammatory and vasoconstrictor effect of angiotensin Π.

Platelet counts in our obese individuals are significantly higher than in non-obese individuals. This finding is consistent with the findings of Kutluturk *et al*. (25) who evaluated the relationship between platelet count and metabolic risk factors of cardiovascular disease in obesity. There is an ongoing debate on whether obesity is accompanied with platelet activation. Higher concentrations of adipose tissue originating inflammatory markers may contribute to atherogenesis and thrombosis through its effects on platelet activation (26). In our study, higher CRP and IL-6 concentrations in obese individuals may be responsible for elevated platelet count in this group.

**T1able 3.** Predictors of WBC in partial correlation analysis (controlling for the effect of physical activity)

Variable	r	p value
Body fat (%)	0.5	<0.001
CRP (mg/L)	0.3	0.05
Ang Π (pg/mL)	0.3	0.049
WC (cm)	0.3	0.02

Ang Π=Angiotensin Π;

CRP=C-reactive protein;

WC=Waist-circumference

The WBC count was associated with triglyceride levels and atherogenic index of plasma as a strong predictor of various clinical and biochemical markers of CVD, especially smaller size of LDL particle (27). This result was consistent with the findings of Ohshita *et al*. (4) and Targher *et al*. (28). They reported that WBC count is associated with components of metabolic syndrome, such as BMI, WHR, and triglyceride. A positive relationship was also found between LDL cholesterol concentrations and haemoglobin or haematocrit levels. This result agreed with the study of Shimakawa *et al*. (29) and Tomoko *et al*. (30) reporting strong positive relationship between serum total cholesterol and haemoglobin (p<0.01 and p<0.002 respectively); the authors suggested that haemoglobin and haematocrit are associated with the risk factors of cardiovascular disease.

**T1able 4.** Relationship among haematological parameters and metabolic characteristics

Variable	WBC (×10^9^/L)	HGB (g/L)	HCT (Proportion of 1.0)	PLT(×10^9^/L)
FBS (mmol/L)	0.1 (NS)	-0.1 (NS)	0.1 (NS)	-0.1(NS)
TG (mmol/L)	0.2 (0.03)	-0.1 (NS)	-0.1 (NS)	0.1 (NS)
TC (mmol/L)	0.1 (NS)	0.1 (NS)	0.2 (NS)	-0.1 (NS)
LDL-C (mmol/L)	0.1 (NS)	0.2 (0.05)	0.3 (0.01)	0.1 (NS)
HDL-C (mmol/L)	0.1(NS)	0.1 (NS)	-0.1 (NS)	0.1 (NS)
AIP	0.3 (0.028)	-0.1 (NS)	-0.1 (NS)	0.2 (NS)

AIP=Atherogenic index of plasma;

FBS=Fasting blood sugar;

HCT=Haematocrit;

HDL-C=High-density lipoprotein cholesterol;

LDL-C=Low-density lipoprotein cholesterol;

NS=Not significant;

PLT=Platelet count;

TC=Total cholesterol;

TG=Triglyceride;

WBC=White blood cell;

Values expressed as r with p values in parentheses

### Limitations

The present study had several limitations. First, the case-control nature of the study lacks information about the causal relationship between variables. This needs to be elucidated in future prospective studies. Second, we did not assess leptin level in our study. It has been suggested that relation of body fat and WBC count may be mediated by leptin (15). Circulating leptin concentrations increase in obesity (31). Several studies reported the *in-vitro* effects of leptin in proliferation of stem cells and WBC production (32) and that leptin potentiates platelet aggregation and activation in obesity (33,34). Therefore, further studies regarding the role of leptin in developing abnormal WBC or platelet production in obesity are desirable. Third, this study was carried out only in women and not in men; therefore, the results may not be generalized for total population.

### Conclusions

In our study, obese women had higher WBC, platelet count, and inflammatory biomarkers compared to non-obese women. This could represent higher risk of disorders associated with cardiovascular disease or metabolic syndrome in obesity. It was also found that WBC was associated with inflammatory markers, suggesting that higher WBC count in obesity may be mediated by inflammatory factors.

## ACKNOWLEDGEMENTS

This work was supported by a grant from Research Undersecretary of Tehran University of Medical Sciences (Grants no. 11869). We are grateful to the study participants who gave their time and efforts.
